# Extraanatomical revascularization via dermatodermal fusion in pediatric proximal fingertip amputations: A cohort study

**DOI:** 10.1016/j.jpra.2026.03.013

**Published:** 2026-03-19

**Authors:** Susanne Deeg, Michael Boettcher, Daniel Svoboda

**Affiliations:** Department of Pediatric Surgery, University Medical Center Mannheim, Heidelberg University, Theodor-Kutzer-Ufer 1-3, 68167 Mannheim, Germany

**Keywords:** Fingertip amputation, Dermatodermal fusion, Children, Extraanatomical revascularization, Infants, Pediatric

## Abstract

**Background:**

Proximal fingertip amputations in zones 3 and 4, according to the Allen classification, pose a significant challenge in children due to the technical difficulty of performing microvascular anastomosis on small vessels. The goal of this study was to evaluate the effectiveness of dermatodermal fusion as an alternative method for revascularizing amputated fingertips in young children.

**Methods:**

In the current study we present a series of 10 children treated with dermatodermal fusion for proximal fingertip amputations with a mean age of 2.9 years [range: 9 months to 7 years] in one center between 2015 and 2024.

**Results:**

The average age of children with fingertip amputations in zones 3 and 4 was 2.9 years. The surgical technique involved partial-thickness dermabrasion of both the amputated fingertip and a donor site, followed by the fusion of these surfaces. Postoperative care included weekly dressing changes, with surgical separation performed once stable reperfusion was achieved, typically within 3–5 weeks. The results indicated a successful revascularization rate of 80%, with full regrowth in 8 out of 10 patients. Younger children demonstrated better outcomes, supporting the hypothesis that age is a significant factor in regenerative capacity.

**Discussion:**

Dermatodermal fusion offers a simple, effective approach to fingertip replantation, particularly when microsurgical resources are not available. Further studies with larger sample sizes are warranted.

## Introduction

Fingertip injuries are a common occurrence in children, frequently resulting from the accidental trapping of fingers in doors, especially on the hinge side. While injuries can also occur from sharp objects like knives or bread slicers, door-related incidents remain the primary cause.^1^ According to the Allen classification, fingertip injuries are categorized into four zones. Zone 1 involves damage to the soft tissue pulp, while zone 2 includes injuries to the pulp and distal nail bed without bone involvement. Zone 3 encompasses injuries extending to the proximal nail bed with bony involvement, and zone 4 injuries extend even further, possibly even to the level of the distal interphalangeal (DIP) joint.^2^

Conservative management, such as sterile film dressings,^3^^,^^4^ is often sufficient for distal fingertip amputations that do not much involve the nail bed or bone.^5^ However, for more proximal injuries, especially those in zones 3 and 4, preserving both the length of the finger and the integrity of the nail bed is crucial. In such cases, replantation becomes desirable. Microvascular anastomosis is the gold standard for restoring perfusion but may be impractical in young children due to the small size of the vessels.^6^^,^^7^ In adults, many different flaps have been described to cover the fingertip with mixed results.^8^ Dermatodermal fusion has been proposed as an alternative technique that does not require microvascular expertise, providing a simpler yet effective method for extraanatomical revascularization.

The technique, initially described by Vilkki,^9^ involves creating an avascular connection between the amputated fingertip and a donor site, allowing the graft to revascularize through dermatodermal fusion. This study aims to present our experience with this technique in a pediatric cohort and evaluate its outcomes.

## Methods

In this retrospective study all patients presenting at our center with proximal fingertip amputations and treatment with the dermatodermal fusion technique between 2015 and 2024 were included.

A database search was carried out for the surgical procedure of finger replantation, after which the cases with dermatodermal fusion were filtered out.

Those cases were evaluated concerning the following criteria: Gender, age at the time of injury, mechanism of injury, injured finger, side (right/left), type of surgical treatment, duration of surgery, duration until surgical separation of the dermatodermal fusion, take rate of the replant, duration of follow-up.

## Dermatodermal fusion

**Figure 1:**
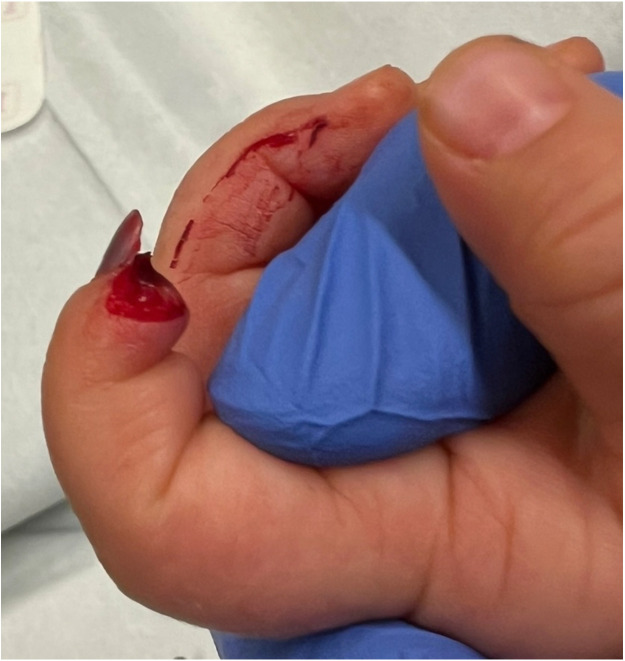
Fingertip amputation.

**Figure 2:**
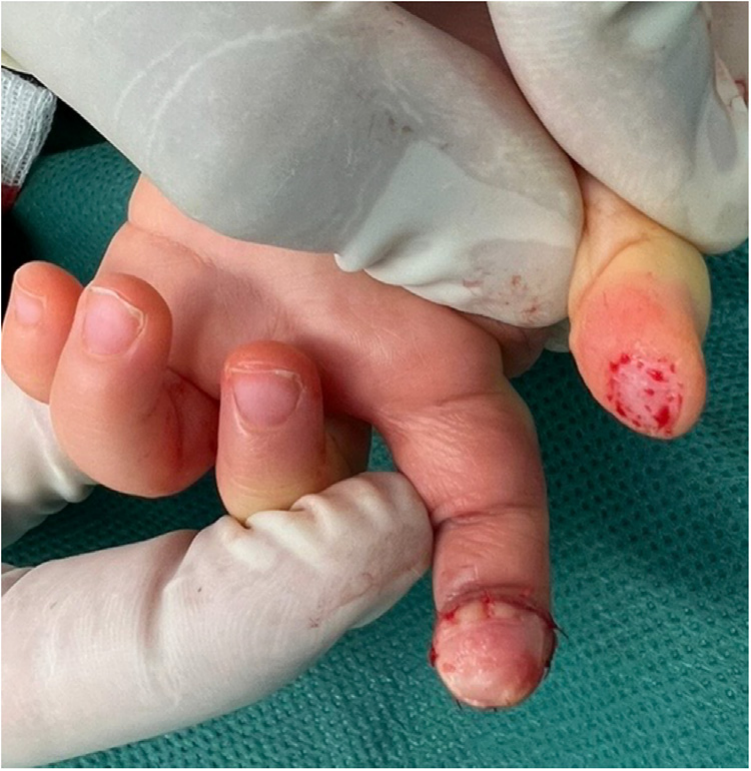
Partial-thickness dermabrasion.

**Figure 3:**
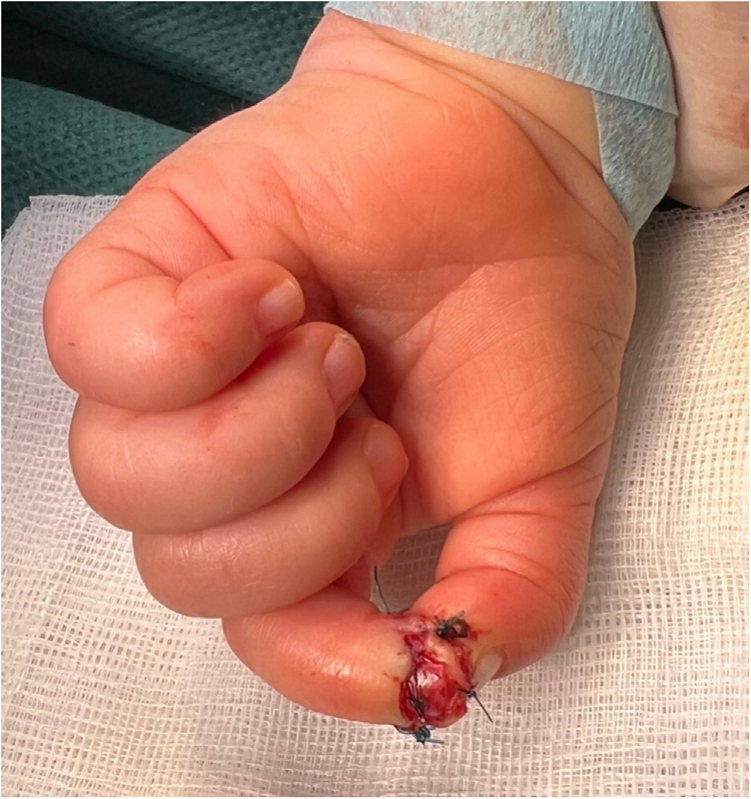
Dermatodermal fusion.

The dermatodermal fusion technique was developed as a straightforward and accessible approach to revascularize proximal amputated fingertips [Figure 1], particularly in settings where microvascular surgery is not feasible.^7^ The procedure begins with meticulous cleaning of the amputated fingertip and composite-grafting. In cases where significant bone involvement is present, stabilization with Kirschner wires (0.6 mm or 0.8 mm) with or without temporary DIP-joint-arthrodesis is performed. Next, partial-thickness dermabrasion using the Goulian Weck skin graft knife is carried out on both the amputated fingertip and the donor site (either the thenar region or the thumb tip or tip of an opposite finger) down to the corium layer (operative endpoint: punctate bleeding at the donor site and yellowish-white color at the amputate side) [Figure 2]. The prepared surfaces are brought into close contact and sutured with robust, non-resorbable button sutures to ensure stable contact and promote revascularization from the donor site [Figure 3]. Surgery is terminated with dry dressing using the following dressing layers: basic layer consisting of a silicone sheet or fatty gauze, second layer consisting of compresses and a compress roll. Topical antibiotics or splinting are not necessary.

**Figure 4:**
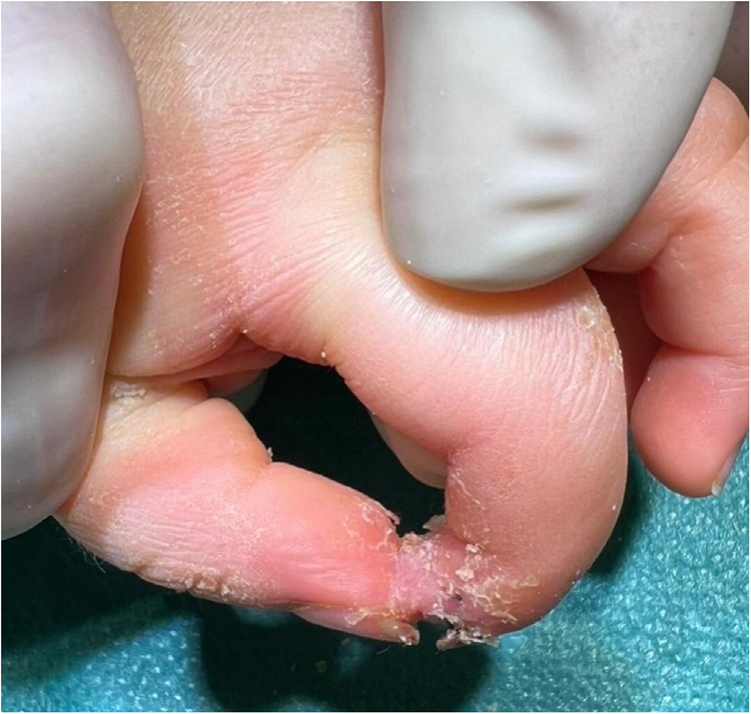
Stable reperfusion.

Postoperative management involves weekly dressing changes to monitor the graft’s viability. Surgical separation of the fused tissue is performed after signs of stable reperfusion, typically after 3–5 weeks [Figure 4].

## Results

In the study period 10 children were surgically treated with dermatodermal fusion for proximal fingertip amputations. The patients age ranged from 9 months to 7 years, with a mean age of 2.9 years. The cohort consisted of nine boys and one girl.

Injury distribution was skewed towards the left hand (8 cases) compared to the right hand (2 cases). Specific digit involvement included the index finger (DII) in 5 cases, the middle finger (DIII) in 2 cases, and the little finger (DV) in 3 cases. According to the Allen classification, there were eight cases classified as zone 3, one as zone 4, and one complex avulsion injury involving detachment of both arteries (A3 and A4) with an unstable open proximal middle phalanx fracture [Table 1]. For all patients, the distal phalanx was reimplanted as a composite graft with nail bed suturing. In 5 patients, osteosynthesis was performed using Kirschner wires, with 4 cases also involving temporary DIP joint arthrodesis. As illustrated in Table 1, Kirschner wires were used when the distal bone fragment was relatively large, namely in two Allen 4 cases and in two elderly children (age 6 years) with Allen 3 injury. Epineural sutures were performed in two elderly children (6 years and 7 years) with larger anatomical structures and in one without severe crush damage where epineural suture was well feasible.

**Table 1:** Table with individual patients’ key data.

Age	Finger	Left/Right	Allen Zone	Succes	OP
2 y	DIII	L	3	Yes	Vilkki
2 y	DII	L	3	Yes	Vilkki + epineural sutures N3 + N4
1 y	DII	L	4	Yes	Vilkki + *K*-wire
6 y	DII	L	3	80% Yes	Vilkki + *K*-wire + epineural suture N3
7 y	DV	L	4	Yes	Vilkki + epineural suture
1 y	DV	L	3	Yes	Vilkki
3 y	DII	R	3	Yes	Vilkki
9 mo	DII	L	3	Yes	Vilkki
1 y	DIII	R	3	Yes	Vilkki
6 y	DV	L	3	No	Vilkki + *K*-wire

The donor site for extraanatomical revascularization was either the thenar or hypothenar region (5 cases) or the thumb tip (5 cases). Mean duration of surgery amounted 50 min [range: 21 min. to 66 min.].

**Figure 5:**
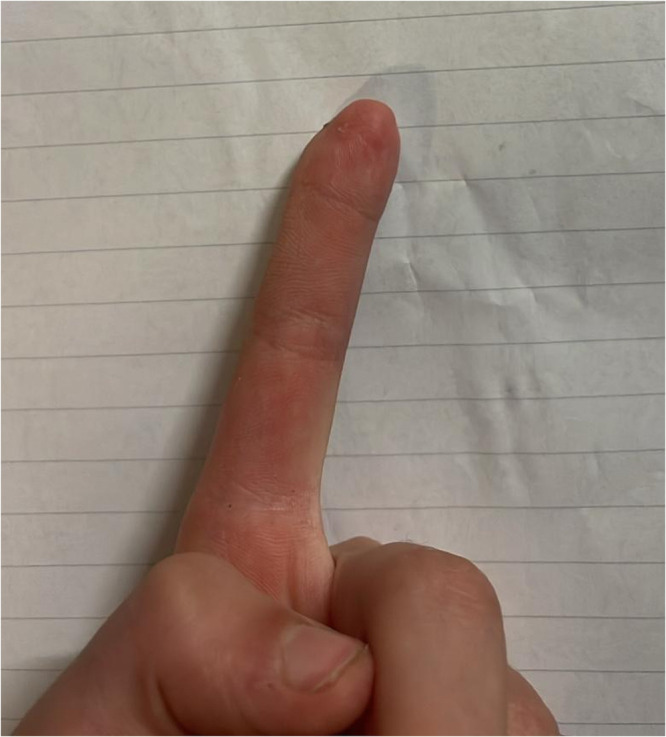
Final condition DII injury, palmar aspect.

**Figure 6:**
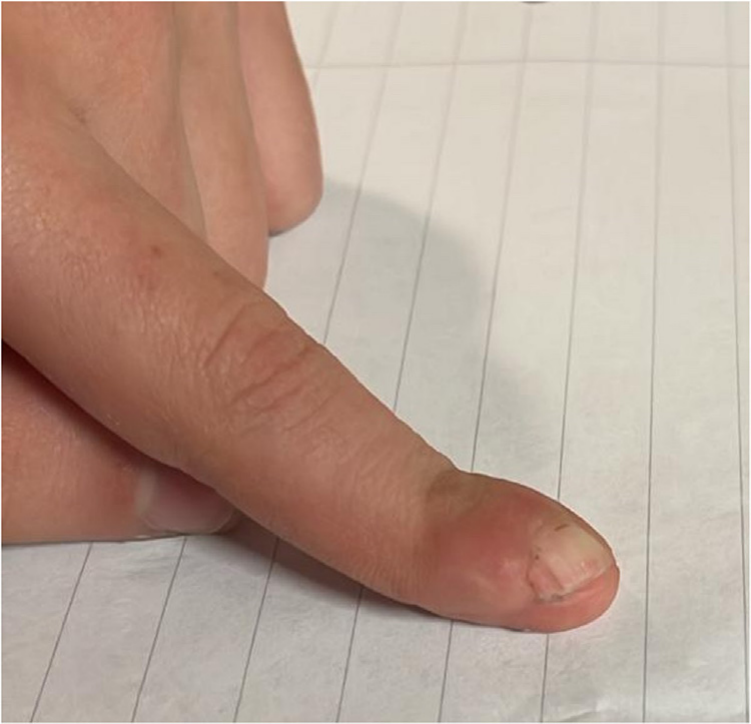
Final condition DII injury, dorsal aspect.

**Figure 7:**
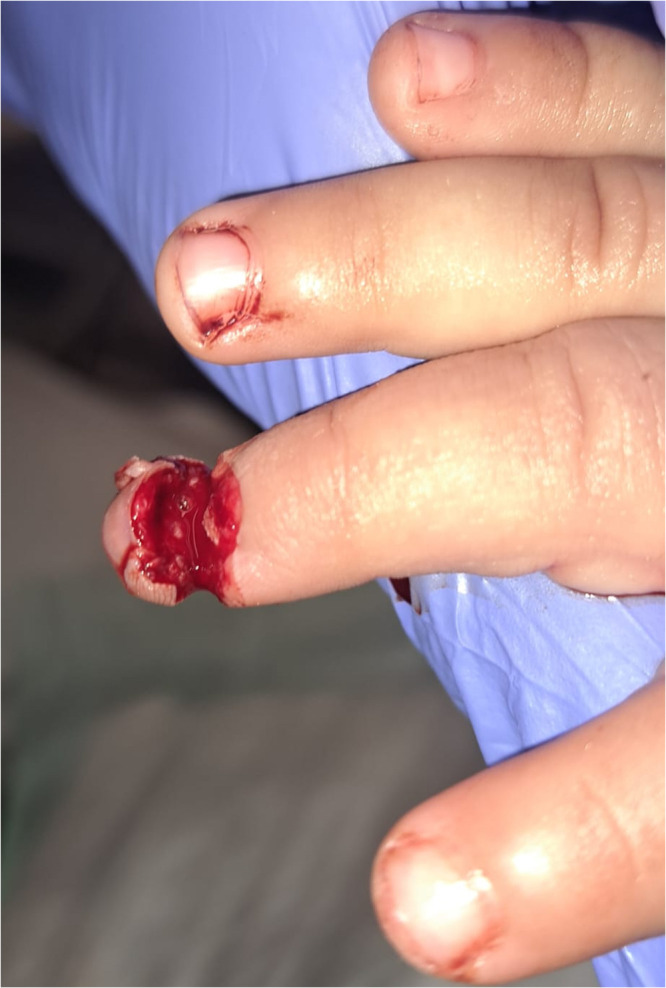
Primary injury DIII.

**Figure 8:**
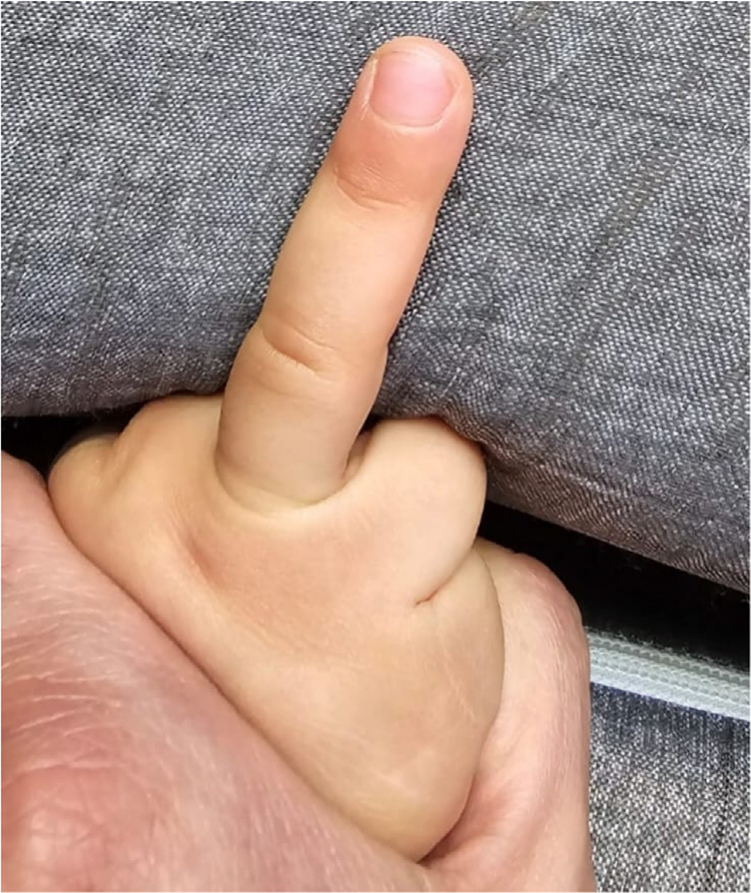
Final condition DIII injury, dorsal aspect.

**Figure 9:**
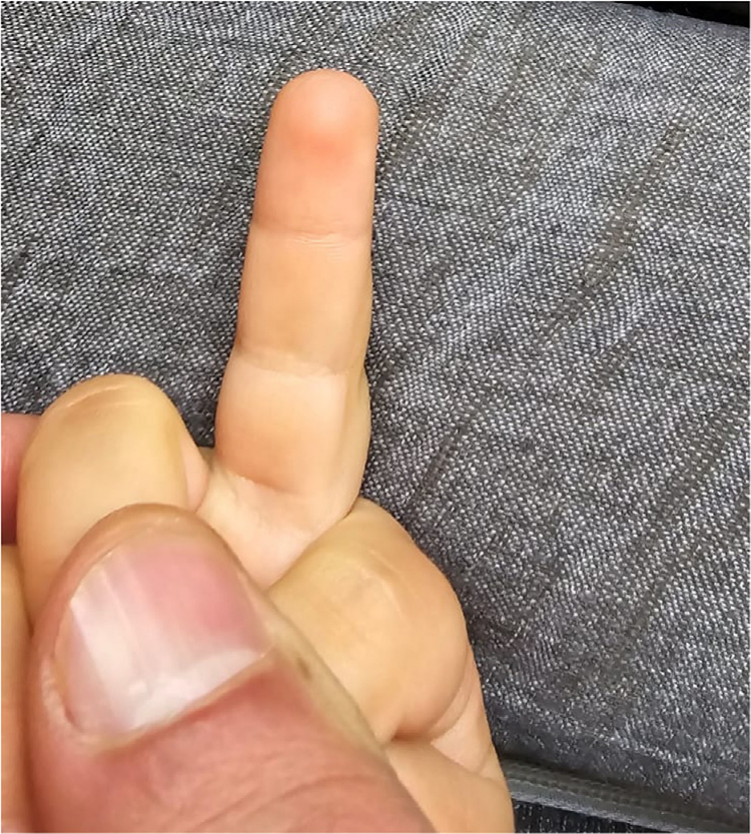
Final condition DIII injury, palmar aspect.

**Figure 10:**
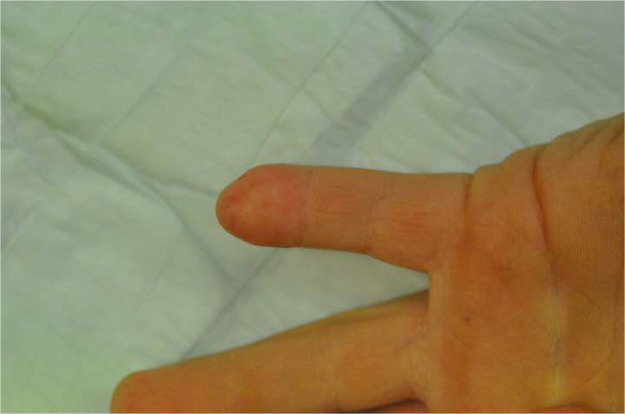
Final condition DV injury, palmar aspect.

**Figure 11:**
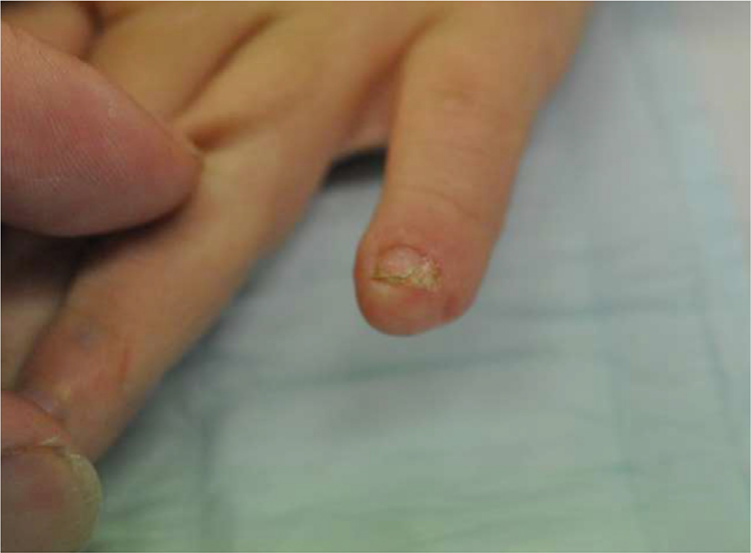
Final condition DV injury, dorsal aspect.

**Figure 12:**
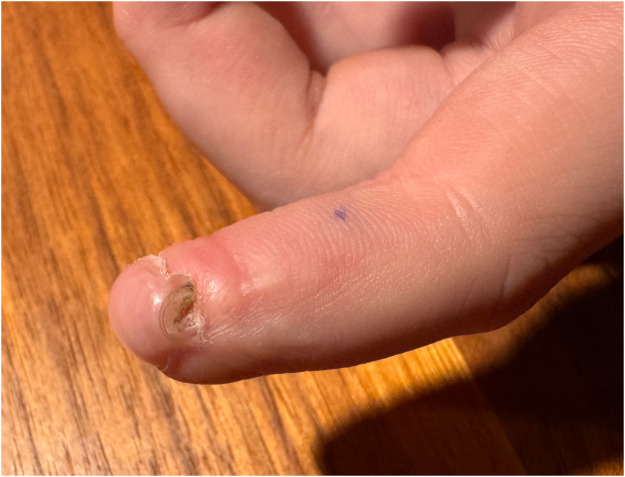
Round nail DII, final condition, palmar aspect.

**Figure 13:**
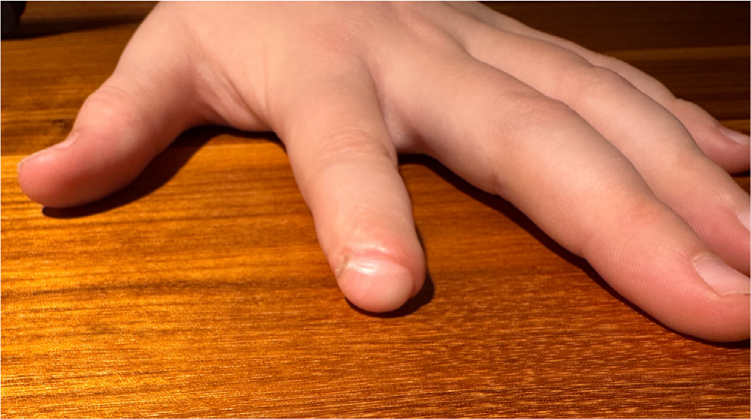
Round nail DII, final condition, dorsal aspect.

Surgical separation of dermatodermal fusion was performed after visible signs of stable reperfusion, after an average time of 25,7 days [range: from 19 to 33 days]. Out of the 10 patients treated with dermatodermal fusion, 8 achieved full revascularization and regrowth of the amputated tissue, resulting in an 80% success rate. One patient, a 6-year-old boy with a zone 3 injury on the left digit V, experienced total graft loss despite optimal care. Six months later we saw him again, his little fingertip being shortened but possessing a small nail. Another 6-year-old patient achieved only partial regrowth (approximately 80% of the graft remained intact). Generally, no scarring, stiffness or loss of range of motion were identified at the donor thumb/donor site. We did not note any infection. The injured fingers with successful revascularization showed no or minor nailbed or fingertip deformities [Figure 5, Figure 6, Figure 7, Figure 8, Figure 9, Figure 10, Figure 11]; in the aforementioned case of incomplete restitution, the growth of a round nail was observed [Figures 12 and 13]. There, we plan to proximalize the nail matrix in another procedure.

The average follow-up time was 262 days (range: 24 days to 3.5 years depending on success rate).

## Discussion

### Results

In our retrospective study 10 children (mean age 2.9) with proximal fingertip amputations type Allen 3 or 4 were treated with dermatodermal fusion, which is a technique of additional non-anatomical revascularization without microvascular anastomosis. Stable reperfusion was established in 80% of the cases. No donor site morbidity was noted. In the two cases with complete or partial graft loss patient’s age was 6 years. In the successful cases, no functional impairment and no or only minor nail deformities werde noted.

### Interpretation

**Figure 14:**
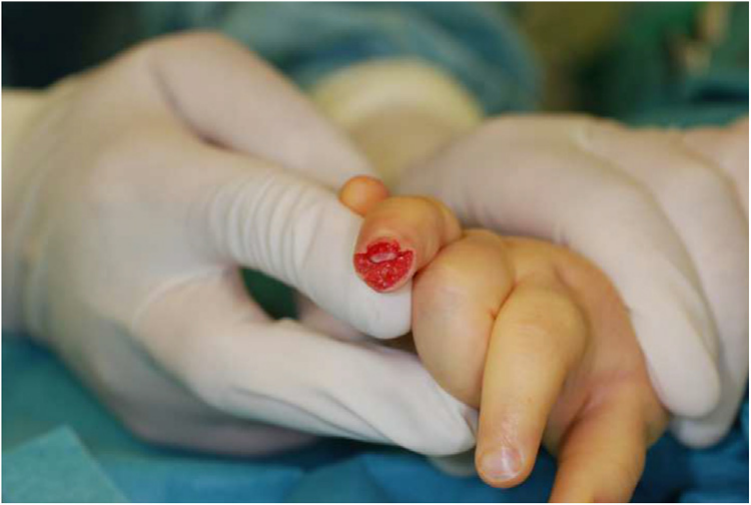
Dermatodermal fusion, donorsite hypothenar: DIV Allen 3 injury.

**Figure 15:**
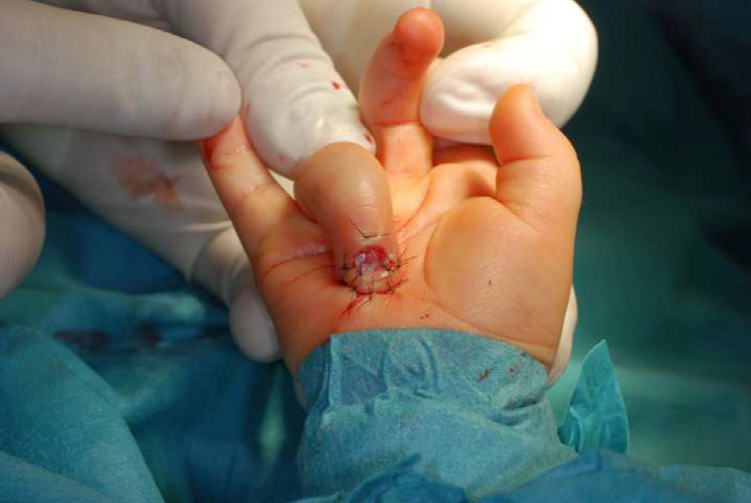
Dermatodermal fusion, donorsite hypothenar: initial procedure.

**Figure 16:**
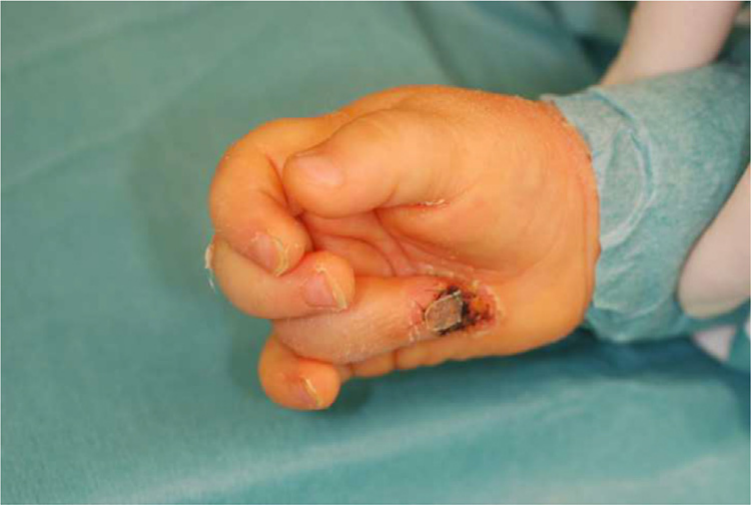
Dermatodermal fusion, donorsite hypothenar: reperfusion.

**Figure 17:**
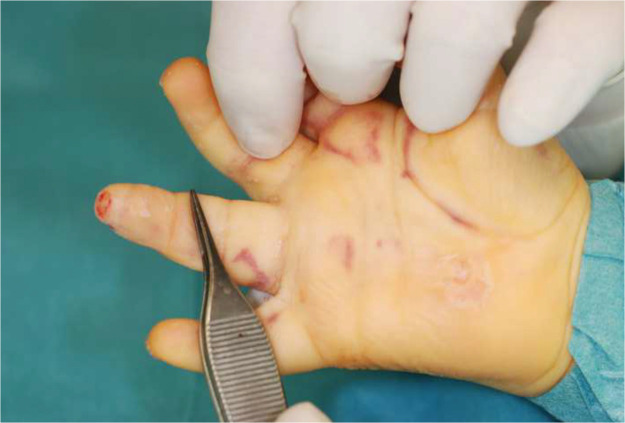
Dermatodermal fusion, donorsite hypothenar: separation.

The findings of this study demonstrate that dermatodermal fusion is a viable alternative to microvascular surgery for treating proximal fingertip amputations in children. At the beginning thenar or hypothenar eminence was used as donor site [Figure 14, Figure 15, Figure 16, Figure 17] to maintain free movement of the other fingers. But since there was not seen any donor morbidity, we switched to the thumb tip as donor within the study period, as this put less tension on the suture.

We attribute the two cases with poor results to the higher patient age and not to technical treatment differences. The observation that younger children achieved better outcomes in our study aligns with existing literature on tissue regeneration in infants.^10^

All in all there was no additional damage through surgery; the method simply offered the chance of a higher survival rate for the replanted tissue by enabling perfusion from two sides.

The high success rate of 80% in our cohort indicates that the presented technique can be an effective solution in settings where microsurgical expertise and feasibility are limited.

### Context and clinical implications

The procedure is relatively straightforward, requiring only basic surgical skills and equipment, and can be performed in a short timeframe.

In comparison, pure composite grafting of pediatric phalanx amputations has shown poorer results in other studies with a complete graft take of between 7 and 17%.^11^, ^12^, ^13^ Possible complications are nail bed deformities, shortening of the fingertip and infections, similar to the potential risks of dermatodermal fusion.

Even if mostly only performed by experts, also microvascular anastomoses can fail.^14^ In a very recent publication, Garcia et al. investigated the outcome of microsurgical anastomoses in the context of finger replantations in children which were performed in one nation between 2004 and 2020. The study included 348 children who were treated in 47 centers with microsurgical expertise. However, the level of amputation was not limited to the distal phalanx. A replantation loss rate of 20% was detected.^15^

The avascular anastomosis reduces the technical difficulty, making dermatodermal fusion an accessible option for surgeons in less specialized settings.

### Limitations

Due to the retrospective nature of this study, no validated scores, questionnaires or objective functional tests relating to hand function had been used during follow-up. Regardless of this, it must be acknowledged that there exist no or only insufficient variants which are not fully suitable for small children. The Massachusetts Hand Outcomes Questionnaire for example would be limited in this study, as no pediatric version has been published yet.

Before all others, the small sample size of this study is a major limitation for the generalizability of our results. Furthermore blinded treatment was no option for those patients. Therefore, a certain observer bias could be present. Future research should aim to include larger patient cohorts and absolutely consider a matched-pair analysis with other conservative treatment options, such as sterile film dressings^4^ or composite grafting. This could hopefully lead to a more reliable causal conclusion regarding which method might be superior to other techniques.

### Conclusion

In conclusion dermatodermal fusion offers a practical and effective approach to managing proximal fingertip amputations in young children. This technique allows for the preservation of finger length and nailbed integrity, with high rates of patient and parent satisfaction. Given its simplicity and effectiveness, it should be considered a valuable option in the armamentarium of pediatric hand surgeons, especially when microsurgical replantation is not feasible.

## Funding source

None.

## Ethical approval

The research protocol was approved by the local Ethical Committee.

## Declaration of generative AI and AI-assisted technologies in the writing process

During the preparation of this work the author used ChatGPT and DeepL in order to improve language and readability. After using this tools/services, the authors reviewed and edited the content as needed and take full responsibility for the content of the publication.

## Declaration of competing interests

None declared.
